# Prognostic Value of Ezrin in Various Cancers: A Systematic Review and Updated Meta-analysis

**DOI:** 10.1038/srep17903

**Published:** 2015-12-03

**Authors:** Jianwei Li, Kuanhai Wei, Hailang Yu, Dan Jin, Gang Wang, Bin Yu

**Affiliations:** 1 Department of Traumatology and Orthopedics, Nanfang Hospital, Southern Medical University, Guangzhou 510515, China; 2 Institute of Genetic Engineering, Southern Medical University, Guangzhou 510515, China

## Abstract

More and more studies have investigated the effects of Ezrin expression level on the prognostic role in various tumors. However, the results remain controversial rather than conclusive. Here, we performed a systematic review and meta-analysis to evaluate the correlation of Ezrin expression with the prognosis in various tumors. the pooled hazard ratios (HR) with the corresponding 95% confidence intervals (95% CI) were calculated to evaluate the degree of the association. The overall results of fifty-five studies with 6675 patients showed that elevated Ezrin expression was associated with a worse prognosis in patients with cancers, with the pooled HRs of 1.86 (95% CI: 1.51–2.31, *P* < 0.001) for over survival (OS), 2.55 (95% CI: 2.14–3.05, *P* < 0.001) for disease-specific survival (DFS) and 2.02 (95% CI: 1.13–3.63, *P* = 0.018) for disease-specific survival (DSS)/metastasis-free survival (MFS) by the random, fixed and random effect model respectively. Similar results were also observed in the stratified analyses by tumor types, ethnicity background and sample source. This meta-analysis suggests that Ezrin may be a potential prognostic marker in cancer patients. High Ezrin is associated with a poor prognosis in a variety of solid tumors.

Ezrin is an important member of the ERM (Ezrin-radixin-moesin) cytoskeleton-associated proteins family, which started to look like a transit protein between membrane proteins and actin filaments^1^,^2^. Nevertheless, recent studies have revealed that Ezrin is an important signaling molecule that is well-documented to be associated with many cellular processes, including cell proliferation, cell adhesion, cell motility, signal transduction and so on^3^,^4^,^5^,^6^, all of those processes play a vital role in tumorigenesis, development, invasion and metastasis in a variety of human malignancies^7^,^8^,^9^,^10^,^11^,^12^,^13^,^14^.

Ever since the first report about the prognosis effect of Ezrin on uveal malignant melanoma in 2001^15^, numerous studies have been considered on investigating the prognostic effects of Ezrin expression in various tumors, such as bladder cancer, non-small cell lung cancer (NSCLC), breast cancer, squamous cell carcinoma of the head and neck (HNSCC), soft tissue sarcomas(STS), Gastric cancer, Osteosarcoma Hepatocellular carcinoma, ovarian carcinoma and so on^16^,^17^,^18^,^19^,^20^,^21^,^22^,^23^,^24^,^25^,^26^,^27^,^28^,^29^, most of which revealed that a poor prognostic outcome stemed from those cancer patients with high Ezrin expression^15^,^16^,^17^,^18^,^19^,^20^,^21^,^22^,^23^,^24^,^25^,^26^,^27^,^28^,^29^,^30^,^31^,^32^,^33^,^34^,^35^,^36^,^37^,^38^,^39^,^40^,^41^,^42^,^43^,^44^,^45^,^46^. However, because of insignificant or opposite results^47^,^48^,^49^,^50^,^51^,^52^,^53^,^54^, the reliability of Ezrin acting as a prognostic biomarker in various malignancies has not been reached consensus. Therefore, the prognostic value of Ezrin in cancer patients remains controversial. In terms of the limits of the single study, as well as in order to better understanding the significance of Ezrin expression in the prognosis of cancer patients, performing a comprehensive meta-analysis to evaluate the published studies is necessary.

In the present meta-analysis, the aim is to assess the correlation between Ezrin expression and the survival outcomes in cancer patients via collecting global related literatures to carry out a systematic analysis.

## Results

### Study characteristics

As shown in Supplementary Figure S1, a total of 299 articles were initially retrieved using the search strategy. After the manual evaluation of title and abstract, 236 articles were excluded because of being irrelevant or duplicate. Among the remaining 63 articles, 19 were further removed due to lack of the essential data about survival outcome. In addition, There were one article^47^ investigated in two different types of intrahepatic cholangiocarcinoma and another one^50^ investigated in two independent patient cohorts, so we considered the data from these studies as an individual separately. Finally, a total of 44 articles including 55 studies were included in the meta-analysis.

The main characteristics of the eligible studies are summarized in Table 1. All of the 55 studies were retrospective in design. The studies enrolled 6,675 cases (ranged from 19 to 487 per study) from the United States, Sweden, China, the United Kingdom, Italy, Spain, Korea, Brazil, Finland, France, Germany and Japan, which evaluated a wide range of carcinomas, including 14 for digestive cancer, 6 for osteosarcoma, 5 for squamous cell carcinoma of the head, 5 for gynecologic cancer, 5 for bladder cancer, 3 for hepatobiliary cancer , 2 for lung cancer, 3 for soft tissue sarcomas and 10 for “other cancers”. Thirty-six studies comprising 5,456 cases reported HRs for OS, 10 studies comprising 1,709 cases for DFS and 9 studies comprising 1,416 cases for DSS/MFS. Tissue samples with formalin-fixed and paraffin-embedded (FFPE) tissues were used in 37 studies, while 18 studies used tissue microarray (TMA). Immunohistochemical method was used in all studies. In addition, the standard of the cut-off values was no uniform in each study, with the values ranged from at least positive to >80% value.

### Mata-analysis Results

**Figure 1:**
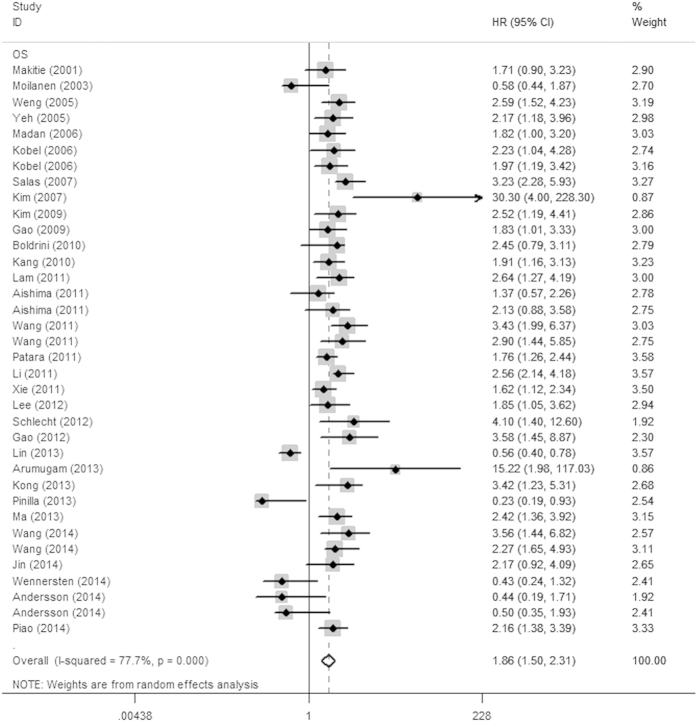
Forrest plots of studies evaluating HRs of Ezrin expression for OS. The squares and horizontal lines correspond to the study-specific HR and 95% CI. The area of the squares reflects the study-specific weight (inverse of the variance). The diamonds represent the pooled HR and 95% CI.

**Figure 2:**
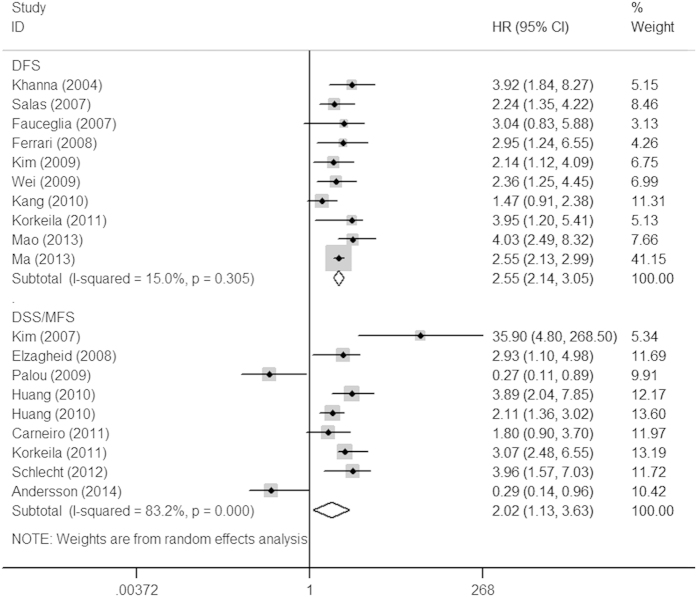
Forrest plots of studies evaluating HRs of Ezrin expression for DFS and DSS/MFS. The squares and horizontal lines correspond to the study-specific HR and 95% CI. The area of the squares reflects the study-specific weight (inverse of the variance). The diamonds represent the pooled HR and 95% CI.

The association between Ezrin expression and various cancers prognosis is illustrated in Fig. 1 and Fig. 2. Overall, elevated Ezrin expression had a worse outcome in cancer patients, with the pooled HRs of 1.86 (95% CI: 1.51–2.31, *P* < 0.001) for OS and 2.02 (95% CI: 1.13–3.63, *P* = 0.018) for DSS/MFS with a random model because of the significant heterogeneity (*I*^2^ = 77.7%, *P* < 0.001; *I*^2^ = 76.7%, *P* < 0.001, respectively). Additionally, high Ezrin expression was also correlated with DFS, with the pooled HR of 2.55 (95% CI: 2.14–3.05, *P* < 0.001) calculated by a fixed model because of the absence of heterogeneity (*I*^2^ = 15%, *P* = 0.305).

To explore the sources of heterogeneity, sub-group analysis for OS and DSS/MFS were conducted by the ethnicity, sample source and cancer types. The main results of this subgroup analysis for prognostic role of Ezrin in various tumors are shown in Table 2. In the ethnicity subgroup analyses, considerable heterogeneity was observed no matter the cancer patients were Asian or Caucasian for OS and DSS/MFS, the results showed that Ezrin over-expression reduced significantly the OS (HR = 2.21, 95% CI:1.72–2.83, *P* < 0.001) and DSS/MFS (HR = 4.18, 95%CI:1.60–10.95, *P* = 0.004) in Asian cancer patients, but not in Caucasian ones (HR = 1.41, 95%CI: 0.95–2.09, *P* = 0.092; HR = 1.40, 95%CI: 0.61–3.19, *P* = 0.426, respectively).

In the sub-group analyses based on sample source, the results demonstrated that high Ezrin expression had a worse prognosis for OS (HR = 2.32, 95% CI:1.84–2.92, *P* < 0.001) and DSS/MFS (HR = 3.82, 95% CI: 2.20–6.64, *P* < 0.001) from FFPE samples, but not those from TMA ones (HR = 1.02, 95%CI: 0.64–1.61, *P* = 0.947; HR = 1.12, 95%CI: 0.46–2.70, *P* = 0806, respectively). However, we founded that there were a significant heterogeneity between the two kinds of samples whether they were for OS or for DSS/MFS.

**Figure 3:**
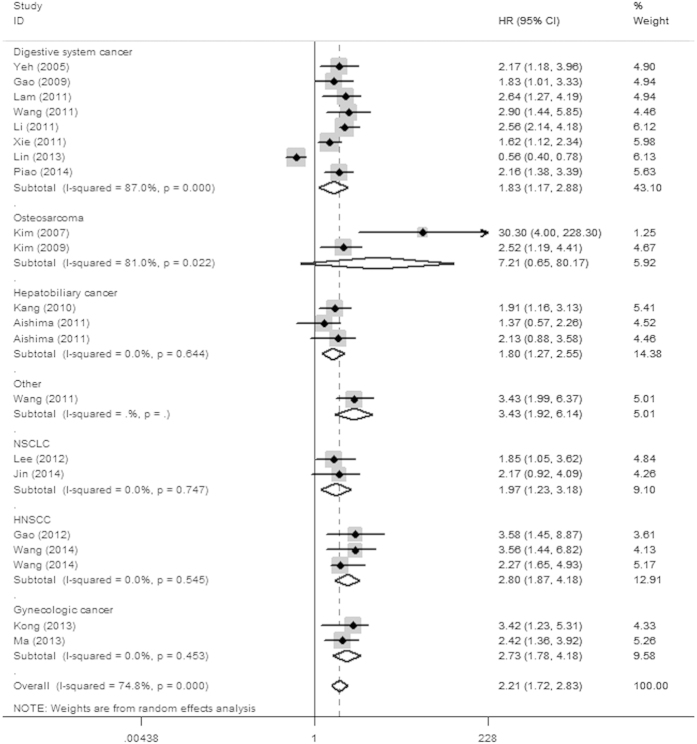
Forest plot of overall survival associated with Ezrin in cancer patients among Asians.

**Figure 4:**
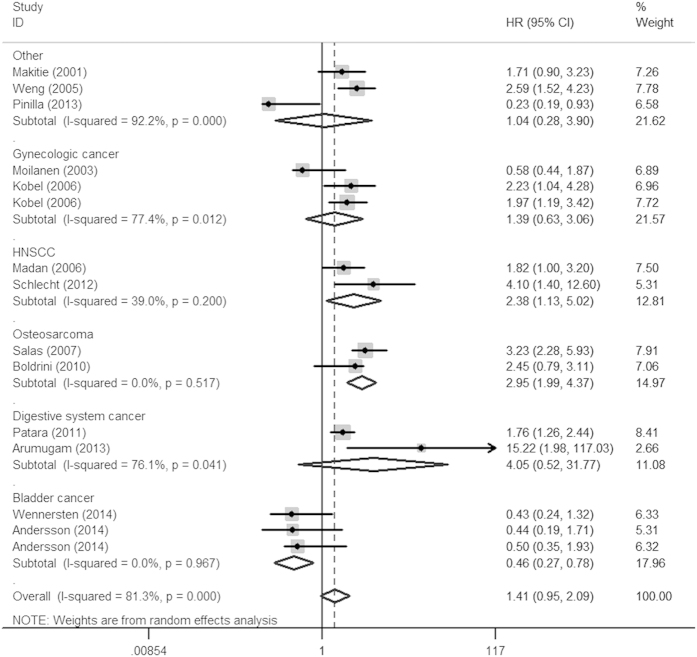
Forest plot of overall survival associated with Ezrin in cancer patients among Caucasians.

In the stratified analyses according to cancer type, over-expression of Ezrin yielded a worse OS in digestive system cancers (HR = 1.93, 95% CI: 1.31–2.85, *P* = 0.001), HNSCC (HR = 2.54, 95% CI: 1.85–3.49, *P* < 0.001), gynecologic cancer (HR = 1.86, 95%CI: 1.10–3.15, *P* = 0.021), osteosarcoma (HR = 3.16, 95% CI: 1.90–5.26, *P* < 0.001), hepatobiliary cancer (HR = 1.80, 95% CI: 1.27–2.56, *P* = 0.001), NSCLC (HR = 1.97, 95% CI: 1.23–3.18, *P* = 0.005) and a worse DSS/MFS in digestive cancers (HR = 3.03, 95% CI: 3.01–4.56, *P* < 0.001). However, positive Ezrin expression was a predictor of good prognosis in bladder cancer for OS (HR = 0.49, 95% CI: 0.27–0.78, *P* = 0.004). Furthermore, we also performed sub-group analysis restricted to cancer type in different ethnicities for OS (Table 3), the results showed that Ezrin positive expression was associated with a poor prognosis of various tumors, especially HNSCC (HR = 2.80, 95% CI: 1.87–4.18, *P* < 0.001) and gynecologic cancer (HR = 2.73, 95% CI: 1.78–4.18, *P* < 0.001) among Asians (Fig. 3), with the exception of osteosarcoma (HR = 7.21, 95% CI: 0.65–80.17, *P* = 0.108). However, individuals elevating Ezrin expression had a significantly improved survival of bladder cancer (HR = 0.46, 95% CI: 0.27–0.78, *P* = 0.004) among Caucasians (Fig. 4).

### Publication bias and sensitivity analysis

**Figure 5:**
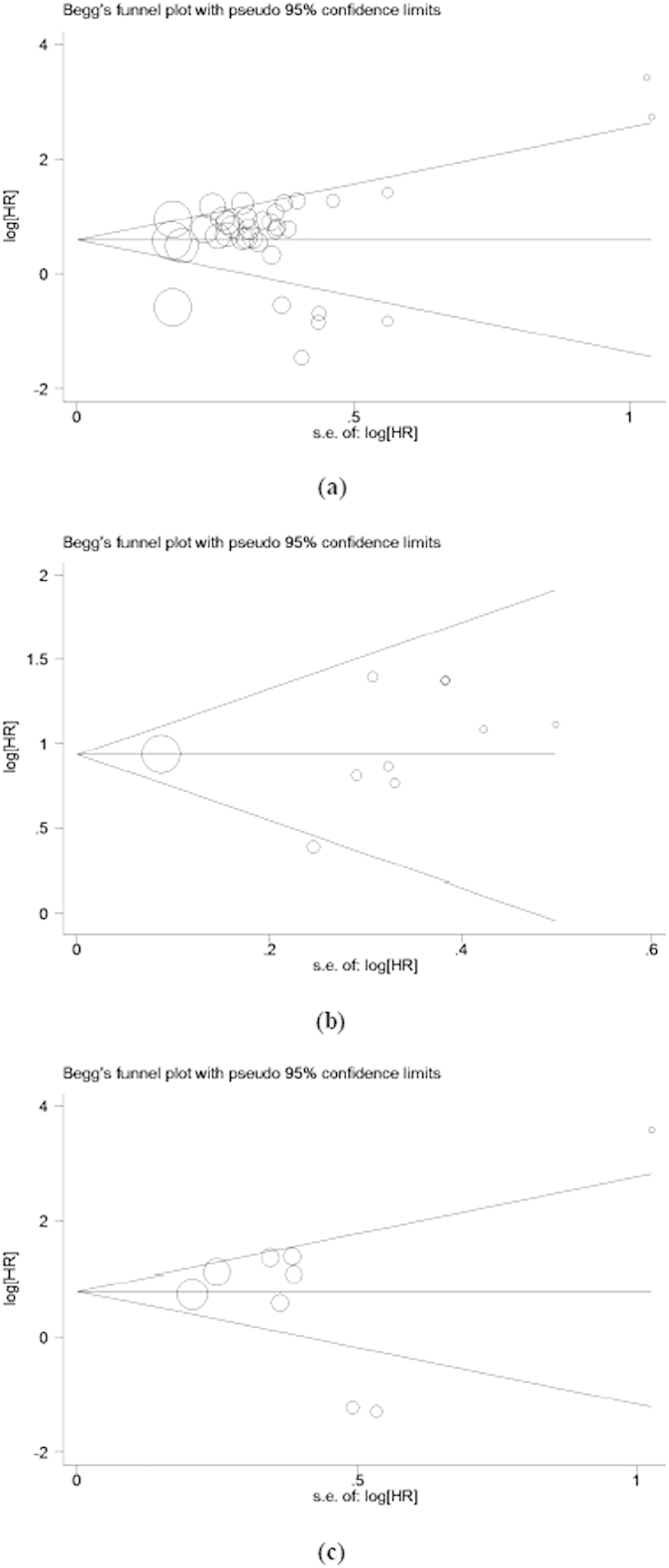
Begg’s funnel plots for publication bias test of OS (**a**), DFS (**b**) and DSS/MFS (**c**).

Both Begg’s funnel plot and the Egger’s test were performed to evaluate the publication bias of the inclusion studies. As shown in Fig. 5a–c, the shape of the funnel plots revealed no obvious asymmetry. And the *P* values of Egger’s test for OS, DFS and DSS/MFS were 0.389, 0.597 and 0.743, respectively, indicating that there was no significant publication bias in the meta-analysis. Meanwhile, the sensitivity analysis was performed to measure the effects of each individual study on the pooled HRs for the OS, DFS or DSS/MFS by omitting studies, respectively. The results demonstrated that no individual study significant influenced the overall HR, as shown in Supplementary Figure S2a, Figure S2b and Figure S2c. This suggested that the results of the present meta-analysis are credible.

## Discussion

Ezrin, the most important member of the Ezrin/radixin/moesin (ERM) family, is mainly expressed in a variety of malignant tissues which originate from epithelial or non-epithelial cells^55^. Generally, Ezrin is mainly distributed in the cytoplasm with an inactive form, Once activated by threonine and tyrosine phosphorylation, Ezrin would transform into a special active form^56^. The basic biological function of Ezrin is to link transmembrane proteins to actin cytoskeleton^57^,^58^. In addition to acting as a cross-linker, Ezrin is involved in transmission of signals in response to extracellular cues^59^,^60^. The biological pathways associated with Ezrin include protein kinase C, Rho-kinase, NF-*k*B, PI3 kinase/Akt and so on^61^. Moreover, as a metastasis-related oncogene, Ezrin also participate in modulating multiple cellular processes^62^, including the formation of microvilli^63^, maintenance of cell shape^64^, cell-cell adhesion^65^, cell motility and invasion^66^. Hence, it seems that Ezrin might play an important role in the development of cancer. There is growing evidence that Ezrin expression level is associated with tumor progression and dissemination^67^. Numerous epidemiological studies have also assessed the correlation of high Ezrin expression and poor outcome in cancer patients so far, such as digestive system cancer^16^,^17^,^18^,^19^,^20^,^21^,^22^,^23^,^24^,^25^, osteosarcoma^26^,^27^,^28^,^29^,^30^,^31^,^79^,^80^, HNSCC^32^,^33^,^34^,^35^,^36^, gynecologic cancer^37^,^38^,^39^, hepatobiliary cancer^43^ and so on. However, the results about the prognostic value of Ezrin expression in cancer patients remain inconsistent. Some studies reported that up-regulated Ezrin was a negative prognostic factor for survival for cancer patients^15^,^16^,^17^,^18^,^19^,^20^,^21^,^22^,^23^,^24^,^25^,^26^,^27^,^28^,^29^,^30^,^31^,^32^,^33^,^34^,^35^,^36^,^37^,^38^,^39^,^40^,^41^,^42^,^43^,^44^,^45^,^46^, However, other studies showed an opposite result^48^,^50^,^51^,^52^,^78^. To resolve the conflicting issues, we performed a systematic review and meta-analysis on the association between Ezrin expression and prognostic value in cancer patients.

As the first qualitative analysis of Ezrin expression related to survival outcome of various tumors, Han *et al.*^68^ retrieved 29 studies and found that over-expression of Ezrin might be associated with worse prognosis. However, the number of inclusion studies in the analysis was not relatively enough and at least 26 eligible studies were not included in the above meta-analysis, of which 8 studies about osteosarcomas were absolutely not included. Furthermore, the data reported by Han *et al.*^68^ for the study by Jörgren *et al.*^69^ were inconsistent with the data and the conclusion provided by Jörgren *et al.*^69^ in their original article. The HR value reported by Han *et al.*^68^ for OS is 1.89 (95% CI = 1.16–3.10), this suggested that high Ezrin expression was associated with worse prognosis in rectal cancer patients. But after carefully studying the data presented by Jörgren *et al.*^69^, we found Jörgren *et al.*^69^ just provided HR value about LR (local recurrence), not about OS. Moreover, the conclusion by Jörgren *et al.*^69^ showed that Ezrin expression had no impact on overall survival of patients with rectal cancer. Therefore, the conclusion by Han *et al.*^68^ was still being debated and uncertain. In view of this, we performed this updated meta-analysis including 44 articles with 55 studies and elucidated that the high Ezrin expression was significantly associated with poor OS, DFS and DSS/MFS in cancer patients.

This meta-analysis was performed according to the guidelines and recommendations for improving the quality of reporting of medical research such as REMARK^70^ and PRISMA^71^. Estimation of HR using multivariate proportional hazards model was used to evaluate the prognostic significance between ezrin expression and survival outcomes in each study, variables entered into the multivariate analysis mainly included Age, Gender, Tumor size, Tumor grade, TNM tumor stage, Lymph node metastasis, Ezrin expression. These positive factors contributed to the strengths of this meta-analysis.

The evidence included in the present meta-analysis indicated Ezrin expression as a poor prognostic marker in a variety of tumors. However, it should be noted that there are some limitations to the analyses presented here. First, because the number of prognostic studies dealing with each type of cancers was ≤5, the results of the particular carcinomas might be less powerful. Second, English articles were only recruited, and language bias might exist. Third, some HRs were calculated indirectly by the data extracted from the literature, however, these data were less reliable than direct data from the original literature. Fourth, different cutoffs used to assess high Ezrin level in the studies might also have contributed to the heterogeneity, because there is not a standard cutoff value of Ezrin level for increased survival risk. Fifth, significant heterogeneity existed in between studies, even though we calculated the pooled subgroup data with random-effects models. The heterogeneity in these studies could be attributed to the differences by different population characteristics or study designs. In addition, different sample types could also explained the heterogeneity, because tissue microarray (TMA) probably obtained more false-negative cases than the whole section. Finally, some inevitable publication bias might exist in the literature-based analysis, because more positive results tended to be published, thus potentially exaggerating the association between Ezrin expression and poor outcomes. Moreover, because all of the included studies were retrospective, which may have also introduced reporting bias. Therefore, our findings should be interpreted with caution.

In summary, our meta-analysis has demonstrated that the high Ezrin expression is significantly associated with poor survival in cancer patients. However, our results should be also considered cautiously for the above reasons. Further multicenter prospective studies and large clinical investigations should be conducted to validate the prognostic value of Ezrin in various tumors.

## Methods

### Search strategy

Guided by the guidelines of the Meta-analysis of Observational Studies in Epidemiology group (MOOSE), we carried out the meta-analysis^72^. A comprehensive search for all relevant articles published until 31 January 2015 that assessed on the prognostic value of Ezrin in various cancers was performed. The PubMed and EMBASE databases were retrieved with the following search terms or keywords:“Ezrin”, “prognosis OR prognostic OR survival OR outcome” and “cancer OR tumor OR carcinoma OR neoplasm”. Human studies were only restricted in this search. In addition, we also manually reviewed the references of relevant articles to obtain additional findings.

### Inclusion and Exclusion Criteria

In this meta-analysis, the candidate studies were recruited according to the following criteria: (i) studied the patients who suffering from any type of cancers; (ii) evaluated Ezrin expression using Immunohistochemical method; (iii) assesed the correlation between Ezrin expression level and clinical outcome; and (iv) English articles. Articles were excluded based on any of the following criteria: (i) reviews, letters, comments, conference abstracts, or laboratory articles; (ii) articles not in English; (*iii*) absence of key information, such as HR, 95% CI, and *P* value, or useful data for calculation established by *Parmar*, *Williamson*, and *Tierney*^73^,^74^,^75^; and (iv) overlapping studies. The most recent or complete studies were selected if the same patient cohort was utilized in different articles. Full manuscript was available after examining the abstract if any doubt of suitability remained as well.

### Quality Assessment

According to a critical review checklist of the Dutch Cochrane Centre proposed by MOOSE, we strictly assessed the quality of all the studies included^72^: (i) a detailed description about study population and origin of country; (ii) a definite description of the study design; (iii) a definite type of carcinoma; (iv) a definite description of outcome assessment; (v) a definite measurement method of Ezrin and (*vi*) a definite cut-off of Ezrin. Otherwise, We would exclude the studies in order to ensure the quality of the meta-analysis.

### Data Extraction and Conversion

Two reviewers extracted the required information from all eligible studies independently. The extracted data included the following elements: the first author’s name, publication year, country of origin, sample size, tumor type, Ezrin measurement method, cut-off value, follow-up duration, the HRs of Ezrin for OS, DFS or DSS/MFS, as well as their 95% CIs and *P* values. Multivariate Cox proportional hazards regression analysis was used in the present analysis. If the HR and its 95% CI were not available directly, they were calculated from the corresponding data or Kaplan-Meier curves provided in the articles using the method reported previously^75^.

### Statistical analysis

All these HRs and the corresponding 95% CIs were calculated to combine the pooled data following *Tierney*’s method^75^. A test of heterogeneity of combined HRs was performed using Cochran’s Q test and Higgin’s*I*^2^ statistics^76^. A *P* value < 0.05 and/or I^2^ > 50% indicated significant heterogeneity, a random-effect model was used to calculate the pooled HR; otherwise, the fixed-effect model was used. Generally, pooled HR of >1 was assumed to indicate a significant association with worse prognosis and was interpreted as statistically significant if the 95% CI for the pooled HR did not overlap one. Sensitivity analysis was carried out by removing each study at a time to evaluate the stability of the results. Publication bias was analyzed by performing funnel plots qualitatively, and estimated by Begg’s and Egger’s test quantitatively. Two sided *P* < 0.05 was considered statistically significant^77^. All analyses used in the meta-analysis were performed by SPSS version 13.0 and STATA version 12.0 (Stata Corp., College Station, TX, USA).

## Additional Information

**How to cite this article**: Li, J. *et al.* Prognostic Value of Ezrin in Various Cancers: A Systematic Review and Updated Meta-analysis. *Sci. Rep.* **5**, 17903; doi: 10.1038/srep17903 (2015).

## Supplementary Material

Supplementary Information

## Figures and Tables

**Table 1:** Main characteristics of the eligible studies included in the meta-analysis.

Author	Year	Origin of population	No. of patients	Type	Sample source	Assay	Positive(n)	Cut-off	Survival analysis	HR estimation	HR(95%)	follow-up (months)
Wennersten	2014	Sweden	263	Bladder cancer	TMA	IHC	112	≥10%	OS	SC	0.43(0.24–1.32)	NA
Andersson	2014	Sweden	100	Urothelial bladder cancer	TMA	IHC	59	﹥17.5%	OS	SC	0.44(0.19–1.71)	71.04(0.36–98.5)
Andersson	2014	Sweden	342	Urothelial bladder cancer	TMA	IHC	120	﹥27.5%	OS	SC	0.50(0.35–1.93)	≥60
					TMA	IHC	136	﹥12.5%	DSS	SC	0.29(0.14–0.96)	
Piao	2014	China	106	PDAC	FFPE tissues	IHC	73	﹥25%	OS	Reported	2.16(1.38–3.39)	NA
Jin	2014	China	108	NSCLC	FFPE tissues	IHC	71	≥25%	OS	SC	2.17(0.92–4.09)	>60
Wang	2014	China	60	LSCC	FFPE tissues	IHC	45	≥50%	OS	SC	2.27(1.65–4.93)	58.1(26–83)
Wang	2014	China	63	TSCC	FFPE tissues	IHC	34	﹥30%	OS	SC	3.56(1.44–6082)	NA
Lin	2013	China	186	CRA	FFPE tissues	IHC	114	at least moderate	OS	Reported	0.56(0.40–0.78)	60
Mao	2013	China	107	brain astrocytomas	FFPE tissues	IHC	96	≥50%	DFS	SC	4.03(2.49–8.32)	2–56
Arumugam	2013	UK and Italy	76	CAV	FFPE tissues	IHC	42	at least positive	OS	Reported	15.22(1.98–117.03)	median 20 m
Kong	2013	China	51	Early–stage cervical cancer	FFPE tissues	IHC	34	﹥25%	OS	SC	3.42(1.23–5.31)	
Pinilla	2013	Spain	117	PTCLs	TMA	IHC	92	﹥80%	OS	SC	0.23(0.19–0.93)	23.44(0–150)
Ma	2013	China	487	Breast cancer	FFPE tissues	IHC	74	≥75%	OS	Reported	2.42(1.36–3.92)	64.8
					FFPE tissues	IHC		≥75%	DFS	Reported	2.55(2.13–2.99)	
Schlecht	2012	USA	130	HNSCC	FFPE tissues	IHC	34	≥10%	OS	Reported	4.10(1.40–12.60)	52.4
					FFPE tissues	IHC		≥10%	DSS	SC	3.96(1.57–7.03)	
Lee	2012	Korea	112	NSCLC	FFPE tissues	IHC	33	at least positive	OS	Reported	1.85(1.05–3.62)	23(1–153)
Gao	2012	China	216	LSCC	FFPE tissues	IHC	129	≥50%	OS	Reported	3.58(1.45–8.87)	65(4–126)
Carneiro	2011	Sweden	227	STS	TMA	IHC	110	at least positive	MFS	Reported	1.80(0.90–3.70)	48(12–228)
Lam	2011	HongKong	150	Gastric cancer	TMA	IHC	117	at least moderate	OS	SC	2.64(1.27–4.19)	NA
Aishima	2011	Japan	41	ICC–Perihilar	FFPE tissues	IHC	20	﹥11%	OS	SC	1.37(0.57–2.26)	37.56
Aishima	2011	Japan	69	ICC–Peripheral	FFPE tissues	IHC	14	﹥11%	OS	SC	2.13(0.88–3.58)	37.56
Wang	2011	China	200	nasopharyngeal carcinoma	FFPE tissues	IHC	134	at least moderate	OS	SC	3.43(1.99–6.37)	76.8(10.3–117.5)
Wang	2011	China	75	SACC	FFPE tissues	IHC	23	at least intense	OS	SC	2.90(1.44–5.85)	99.37(52–138)
Patara	2011	Brazil	250	CRA	TMA	IHC	21	at least moderate	OS	SC	1.76(1.26–2.44)	NA
Li	2011	China	436	Gastric cancer	TMA	IHC	236	at least moderate	OS	SC	2.56(2.14–4.18)	﹥60
Korkeila	2011	Finland	76	Rectal cancer	FFPE tissues	IHC	33	at least moderate	DFS	SC	3.95(1.20–5.41)	40(2–113)
					FFPE tissues	IHC		at least moderate	DSS	SC	3.07(2.48–6.55)	
Xie	2011	China	307	ESCC	TMA	IHC	240	at least moderate	OS	Reported	1.62(1.12–2.34)	NA
Boldrini	2010	Brazil	34	osteosarcomas	FFPE tissues	IHC	26	≥50%	OS	AP/ED	2.45(0.79–3.11)	27.4(9–69)
Huang	2010	Taiwan	74	Myxofibrosarcomas	TMA	IHC	35	at least moderate	DSS	SC	3.89(2.04–7.85)	53.7
					TMA	IHC		at least moderate	MFS	SC	2.11(1.36–3.02)	
Kang	2010	Korea	100	Hepatocellular carcinoma	FFPE tissues	IHC	28	﹥10%	OS	Reported	1.91(1.16–3.13)	82(41–162)
					FFPE tissues	IHC		﹥10%	DFS	Reported	1.47(0.91–2.38)	
Wei	2009	Taiwan	347	GISTs	TMA	IHC	229	≥50%	DFS	Reported	2.36(1.25–4.45)	36.6(1–235)
Palou	2009	Spain	92	Bladder tumors	TMA	IHC	12	﹥20%	DSS	SC	0.27(0.11–0.89)	90.5(3–173)
Kim	2009	Korea	70	osteosarcoma	FFPE tissues	IHC	39	﹥10%	OS	SC	2.52(1.19–4.41)	59.9
					FFPE tissues	IHC		﹥10%	DFS	SC	2014(1.12–4.09)	
Gao	2009	China	193	ESCC	FFPE tissues	IHC	90	≥50%	OS	SC	1.83(1.01–3.33)	65(4–126)
Elzagheid	2008	Finland	74	Colorectal cancer	FFPE tissues	IHC	61	at least moderate	DSS	SC	2.93(1.10–4.98)	30.8(4.7–149.8)
Ferrari	2008	Italy	95	osteosarcomas	FFPE tissues	IHC	76	at least positive	DFS	SC	2.95(1.24–6.55)	47(10–115)
Fauceglia	2007	USA	108	HNSCC	TMA	IHC	93		DFS	AP/DE	3.04(0.83–5.88)	
Kim	2007	Korea	64	osteosarcomas	FFPE tissues	IHC	33	at least positive	OS	Reported	30.30(4.00–228.30)	78.2(12–137)
					FFPE tissues			at least positive	MFS	Reported	35.90(4.80–268.50)	
Salas	2007	France	37	osteosarcomas	FFPE tissues	IHC	23	﹥1%	OS	SC	3.23(2.28–5.93)	54(10–150)
					FFPE tissues	IHC		﹥1%	EFS	SC	2.24(1.35–4.22)	
Madan	2006	USA	40	HNSCC	FFPE tissues	IHC	19	≥10%	OS	Reported	1.82(1.00–3.20)	41.2(1–128)
Köbel	2006	Germany	164	Endormetrioid carcinomas	FFPE tissues	IHC	83	at the median	OS	SC	2.23(1.04–4.28)	57.4(0.13–93.4)
Köbel	2006	Germany	105	ovarian carcinoma	FFPE tissues	IHC	51	at least moderate	OS	SC	1.97(1.19–3.42)	37.3(1.13–96.5)
Weng	2005	Sweden	50	STS	FFPE tissues	IHC	25	﹥1%	OS	SC	2.59(1.52–4.23)	90(50–134)
Yeh	2005	Taiwan	84	Pancreatic cancer	FFPE tissues	IHC	49	at least moderate	OS	SC	2.17(1.18–3.96)	NA
Khanna	2004	USA	19	osteosarcomas	TMA	IHC	9		DFS	SC	3.92(1.84–8.27)	NA
Moilanen	2003	Finland	440	ovarian carcinoma	TMA	IHC	318	≥10%	OS	SC	0.58(0.44–1.87)	152.4
Mäkitie	2001	Finland	130	Uveal Malignant Melanoma	FFPE tissues	IHC	83	at least positive	OS	Reported	1.71(0.90–3.23)	264(216–312)

TSCC: tongue squamous cell carcinoma; CRA: colorectal adenocarcinoma; SACC: Salivary gland adenoid cystic carcinoma; CAV: cancer of the ampulla of Vater; PDAC: pancreatic ductal adenocarcinoma; NSCLC: nonsmall cell lung cancer; STS: soft tissue sarcomas; LSCC: laryngeal Squamous Cell Carcinoma; TSCC: tTongue squamous cell carcinoma; CRA: colorectal adenocarcinoma; CAV: cancer of the ampulla of Vater; PTCLs: peripheral T-cell lymphomas; HNSCC: squamous cell carcinoma of the head and neck; ICC: intrahepaticcholangiocarcinoma; SACC: salivary gland adenoid cystic carcinoma; ESCC: esophageal Squamous Cell Carcinoma; GISTs: gastrointestinal stromal tumors; FFPE: formalin-fixed, paraffin-embedded; TMA: tissue microarray; IHC: immunohistochemistry; HR: hazard ratio; OS: overall survival; DFS: disease-free survival; DSS: disease-specific survival; MFS: metastasis-free survival; SC: survival curve; AP:author provided; DE: data-extrapolated; NA: not available.95% CI: 95% confidence interval;

**Table 2:** Results of meta-analysis for Ezrin on prognostic effect in cancer patients.

Outcome	Variables	No. of studies	Model	Pooled HR(95%)	Heterogeneity	
					*I*^2^(%)	*P*value
**OS**		**36**	**Random**	**1.86(1.51**–**2.31)**	**77.70%**	**0.000**
	**Cancer type**					
	Digestive system cancer	10	Random	1.93(1.31–2.85)	84.70%	0.000
	HNSCC	5	Fixed	2.54(1.85–3.49)	0%	0.489
	Gynecologic cancer	5	Random	1.86(1.10–3.15)	71.10%	0.000
	Osteosarcoma	4	Random	3.16(1.90–5.26)	47.60%	0.026
	Hepatobiliary cancer	3	Fixed	1.80(1.27–2.56)	0%	0.644
	Bladder cancer	3	Fixed	0.49(0.27–0.78)	0%	0.967
	NSCLC	2	Fixed	1.97(1.23–3.18)	0%	0.747
	Other	4	Random	1.41(0.51–3.91)	90.80%	0.000
	**Ethnicity**					
	Caucasian	15	Random	1.41(0.95–2.09)	81.30%	0.000
	Asian	21	Random	2.21(1.72–2.83)	74.80%	0.000
	**Sample source**					
	FFPE	26	Random	2.32(1.84–2.92)	71.20%	0.000
	TMA	10	Random	1.02(0.64–1.61)	85.50%	0.000
**DFS**		**10**	**Fixed**	**2.55(2.14**–**3.05)**	**15.00%**	**0.305**
	**Cancer type**					
	Osteosarcoma	4	Fixed	2.60(1.90–3.65)	0%	0.605
	Digestive system cancer	2	Fixed	2.92(1.80–4.75)	4.80%	0.305
	Other	4	Random	2.48(1.70–3.60)	58.90%	0.063
	**Ethnicity**					
	Caucasian	5	Fixed	3.02(2.17–4.20)	0%	0.734
	Asian	5	Random	2.37(2.14–3.05)	45.60%	0.119
	**Sample source**					
	FFPE	7	Random	2.49(1.97–3.15)	33.90%	0.169
	TMA	3	Fixed	2.94(1.90–4.54)	0%	0.598
**DSS/MFS**		**9**	**Random**	**2.02(1.13**–**3.63)**	**83.20%**	**0.000**
	**Cancer type**					
	Digestive system cancer	2	Fixed	3.03(2.01–4.56)	0%	0.919
	Bladder cancer	2	Random	0.73(0.11–4.65)	88.50%	0.003
	Soft tissue sarcomas	3	Random	1.43(0.45–4.57)	89.60%	0.000
	Other	2	Random	9.71(1.16–81.04)	75.30%	0.044
	**Ethnicity**					
	Caucasian	6	Random	1.40(0.61–3.19)	86.40%	0.000
	Asian	3	Random	4.18(1.60–10.95)	77.60%	0.000
	**Sample source**					
	FFPE	4	Random	3.82(2.20–6.64)	47.70%	0.125
	TMA	5	Random	1.12(0.46–2.70)	87.40%	0.000

Random-effects model was used when p-value for heterogeneity test < 0.05; otherwise, fixed-model was used. *I*^2^ the percentage of variability in HR attributable to heterogeneity. Abbreviations: HNSCC: squamous cell carcinoma of the head and neck; NSCLC: nonsmall cell lung cancer; FFPE: formalin-fixed, paraffin-embedded; TMA: tissue microarray.

**Table 3:** Stratified analyses of Ezrin on overall survival in cancer patients among Asians and Caucasians.

OS	No. of studies	Model	Pooled HR(95%)	Heterogeneity	
				*I*^2^(%)	*P*value
**Asian**	**21**	**Random**	**2.21(1.72**–**2.83)**	**74.80%**	**0.000**
Digestive system cancer	8	Random	1.83(1.17–2.88)	87.0%	0.000
HNSCC	3	Fixed	2.80(1.87–4.18)	0%	0.545
Gynecologic cancer	2	Fixed	2.73(1.78–4.18)	0%	0.453
Osteosarcoma	2	Random	7.21(0.65–80.17)	81.0%	0.022
Hepatobiliary cancer	3	Fixed	1.80(1.27–2.56)	0%	0.644
NSCLC	2	Fixed	1.97(1.23–3.18)	0%	0.747
Other	1	—	3.43(1.92–6.14)	—	—
**Caucasian**	**15**	**Random**	**1.41(0.95**–**2.09)**	**81.30%**	**0.000**
Digestive cancer	2	Random	4.05(0.52–31.77)	76.10%	0.041
HNSCC	2	Fixed	2.38(1.13–5.02)	39.00%	0.200
Gynecologic cancer	3	Random	1.39(0.63–3.06)	77.40%	0.012
Osteosarcoma	2	Fixed	2.95(1.99–4.37)	0%	0.517
Bladder cancer	3	Fixed	0.46(0.27–0.78)	0%	0.967
Other	3	Random	1.04(0.28–3.90)	92.20%	0.000

Random-effects model was used when p-value for heterogeneity test < 0.05; otherwise, fixed-model was used.*I*^2^ the percentage of variability in HR attributable to heterogeneity. Abbreviations: HNSCC: squamous cell carcinoma of the head and neck; NSCLC: nonsmall cell lung cancer.
