# Supplementary material for: Prognostic Value of Ezrin in Various Cancers: A Systematic Review and Updated Meta-analysis
Source: Sci Rep. 2015 Dec 3;5:17903. doi: 10.1038/srep17903 (PMC4668575; doi:10.1038/srep17903)
Supplement: Supplementary Information [file srep17903-s1.pdf]

Prognostic Value of Ezrin in Various Cancers： A Systematic Review and Updated Meta-analysis

Jianwei Li[1,+], Kuanhai Wei[1,*,+], Hailang Yu[2], Dan Jin[1], Gang Wang[1] & Bin Yu[1]

[1] Department of Traumatology and Orthopedics, Nanfang Hospital, Southern Medical University, Guangzhou 510515, China, [2] Institute of Genetic Engineering, Southern Medical University, Guangzhou 510515, China

* corresponding. weikuanhai@126.com

[+]these authors contributed equally to this work

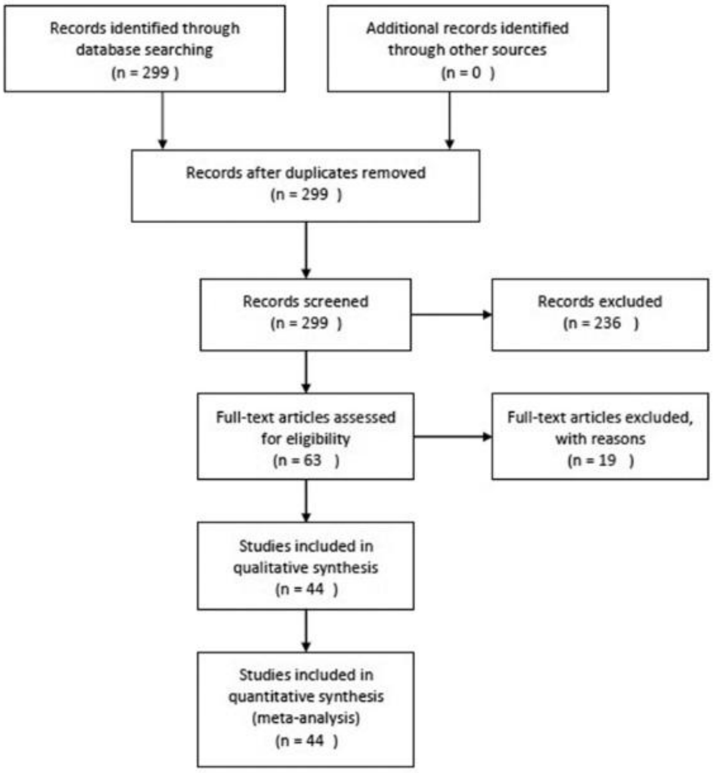

Supplementary Figure S1. Flow chart of literature review and study selection process.

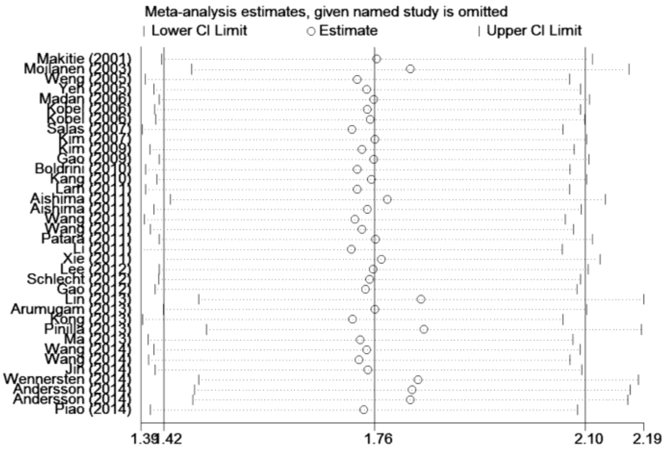

(a)

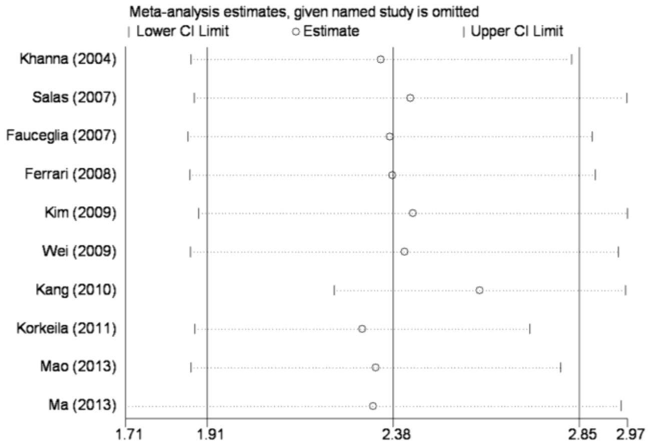

(b)

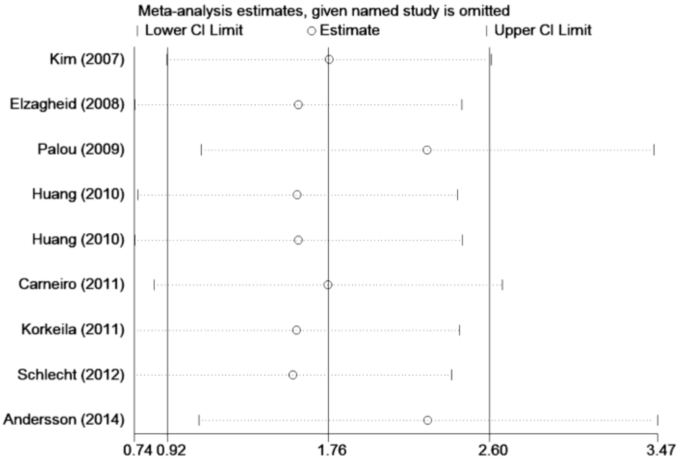

(c)

Supplementary Figure S2. Sensitivity analysis of the influence of individual studies on the summary HR for OS (a), DFS (b) and DSS/MFS (c). The middle vertical axis indicates the overall HR and the two vertical axes indicate its 95% CI. Every hollow round indicates the pooled HR when the left study was omitted in this meta-analysis. The two ends of every broken line represent the 95% CI.
